# Attitudes, perceptions and barriers around evidence-based practice in sports physiotherapy in Kenya

**DOI:** 10.4102/sajp.v77i1.1561

**Published:** 2021-08-30

**Authors:** Thomas K. Mwololo, Benita Olivier, Wallace M. Karuguti, Joseph M. Matheri

**Affiliations:** 1 Department of Physiotherapy, School of Medicine, Jomo Kenyatta University of Agriculture and Technology, Nairobi, Kenya; 2 Department of Physiotherapy, Faculty of Health Sciences, University of the Witwatersrand, Johannesburg, South Africa

**Keywords:** evidence-based practice, standards, sports physiotherapy, Kenya, attitude, perceptions, EBP

## Abstract

**Background:**

Healthcare practitioners are required to integrate clinical experience with the best research evidence for the benefit of the patient.

**Objective:**

Determine the attitudes, perceptions and barriers regarding evidence-based practice (EBP) in sports physiotherapy in Kenya.

**Method:**

A quantitative crosssectional study was conducted among licensed physiotherapists in the Republic of Kenya through a self-administered questionnaire. Associations between selected sociodemographic characteristics (gender, age, training, experience, specialisation) and attitudes, perceptions and barriers were determined using a Chi-square test.

**Results:**

A 55.9% (*n* = 391) response rate was recorded. A positive attitude towards EBP was reported by 94.6% (*n* = 370) of the respondents. The most obvious areas of agreement with attitude-and perception-related statements were that ‘EBP is important in that patients can receive the best possible treatment’ (95.9%; *n* = 375), and that it is important that ‘evidence-based guidelines related to work exist’ (84.6%; *n* = 331). There were no significant associations between the demographic characteristics (gender *p* = 0.104 [*X*^2^ = 2.638;1]; age *p* = 0.495 [*X*^2^ = 2.393;3]; training *p* = 0.590 [*X*^2^ = 4.644;6]; experience *p* = 0.980 [*X*^2^ = 0.426;4] and specialisation *p* = 0.649 [*X*^2^= 0.207;1]); and attitudes and perceptions regarding EBP. Insufficient time was highlighted by 57.8% (*n* = 226) of the respondents as one of the ‘most important barriers’.

**Conclusion:**

Although physiotherapists presented with strong positive attitudes towards EBP in sports physiotherapy, barriers were identified which could hinder the implementation of EBP in sports physiotherapy.

**Clinical implications:**

Barriers to applying EBP in sports physiotherapy may lead to inferior quality of care for athletes while addressing these barriers is crucial.

## Introduction

Healthcare practitioners consider evidence-based practice (EBP) as the gold standard for clinical practice because it supports clinicians in their endeavours to achieve the best patient outcomes with minimal healthcare costs (Herbert et al. 2001).

The Sicily statement on EBP is a statement reflecting the consensus of the delegates at the Second International Conference of Evidence-based Healthcare Teachers and Developers held in Sicily in September 2003. It proposes that the choices of healthcare be based on:

[*T*]he best available current, valid and relevant evidence, made available to those receiving care and informed by the tacit and explicit knowledge of those providing the care within the available resource base. (Dawes et al. 2005)

Furthermore, the components of EBP are defined as: knowledge, skills, attitudes and behaviours (Dawes et al. 2005).

Practitioners in physiotherapy in general, and in sports physiotherapy in particular, are required to integrate clinical experience with the best research evidence for the welfare of the patient (Manske & Lehecka 2012). Clinical experience is the knowledge gleaned from years of experience and training (Herbert et al. 2011).

The practice of sports physiotherapy can be divided into five steps that include: the formulation of a clearly answerable clinical question; tracking down of the best relevant evidence; a critical appraisal of the research evidence for its validity and applicability; the application of the critically appraised research evidence with clinical expertise; and an evaluation of one’s performance, or the outcomes of one’s actions (Condon et al., 2016; Manske & Lehecka 2012). Failure to undertake any of the five steps constitutes a barrier (Condon et al. 2016; Jette et al., 2003; Yahui & Swaminathan 2017).

A substantial commitment to, and a positive attitude regarding EBP are essential for the development of dynamic professionals willing to adhere to the EBP process (Gudjonsdottir et al. 2017). Generally, it has been reported that physiotherapists have positive attitudes towards EBP, and they also recognise the importance of using research to guide their clinical practice (Jette et al. 2003; Scurlock-Evans et al., 2014). A review by Scurlock-Evans et al. (2014), in the United Kindgom (UK), found that most physiotherapists generally have positive attitudes and beliefs regarding EBP. Consensus regarding positive attitudes towards EBP has also been documented in the United States of America (USA) (Jette et al. 2003), both among physiotherapy clinicians and also among clinical instructors in the US universities (Bierwas et al., 2016). The same attitudes have been reported in Sweden, as cited in Nilsagård and Lohse (2010); Heiwe et al. (2011) and Dannapfel et al. (2013). In addition, some countries in the Asia-Pacific region, such as Malaysia (Yahui & Swaminathan 2017), Australia (Iles & Davidson 2006) and India (Panhale & Bellare 2015), have also reported positive attitudes among their respondents. Literature from African countries such as Zimbabwe (Tadyanemhandu et al. 2016) and South Africa (Frantz & Diener 2009) have also reported positive attitudes towards EBP. However, a study in Belgium reported negative attitudes towards the concept, purely as a result of concerns around reduced therapeutic autonomy, with a resultant lack of motivation to implement it (Hannes et al. 2009).

Barriers that interfere with the implementation of EBP have also been reported in various studies and they make it difficult to integrate the EBP model into clinical practice. Two systematic reviews identified ‘lack of time’; ‘poor access to databases’; ‘the inability to make critical appraisals of the literature’ and ‘a lack of understanding of statistical data’ as among the most frequently mentioned barriers (Scurlock-Evans et al. 2014; Silva et al., 2015). In addition, ‘limited journal access’; ‘limited access to online information’ (Iles & Davidson 2006; Maher et al., 2004; Nilsagård & Lohse 2010; Ramírez-Vélez et al. 2015a; Salbach et al., 2009; Yahui & Swaminathan 2017); ‘a lack of employer support’; ‘a lack of resources’; ‘ a lack of interest and misinterpretation of EBP’; ‘the inability to apply research findings to individual patients with unique characteristics’; ‘a lack of research skills’ and ‘a lack of generalisation of results to the specific patient population’, have also been identified as barriers to EBP (Heiwe et al., 2011; Iles & Davidson 2006; Jette et al., 2003). Furthermore, Maher et al. (2004) documented a link between barriers to access and interpretation of evidence with the lack of access to electronic databases (such as MEDLINE, CINHAL and EMBASE), as well as access to the full-text article; and also, to publication language issues. In the African context, barriers to EBP were highlighted in Nigeria by Akinbo et al. (2008), who reported that 37% of the respondents identified ‘difficulties in the application of findings to individual patients having unique characteristics’, as well as ‘poor access to databases’, ‘journal access’ and ‘limited access to online information’.

Like other speciality branches of physiotherapy, sports physiotherapists are required to deliver quality care, and EBP is an important strategy in optimising the quality of care. On the occasions where positive attitudes and a barrier-free clinical environment exist, EBP is a possibility. As EBP is a relatively new concept in Kenya, there is the need to empirically document the attitudes, perceptions and barriers regarding evidence-based sports physiotherapy standards – hence justifying the current study.

## Methods

This cross-sectional descriptive survey was conducted among a total population of 700 physiotherapists licensed to practise by the Physiotherapy Council of Kenya, as of February 2018. There was no exclusion criterion in this study. A census was conducted among the entire total population of 700 physiotherapists. A response rate of 254 participants was considered representative for generalisation purposes (Yamane 1967).

Self-administered survey questionnaires were distributed via email, or hand delivered to those physiotherapists who had been recruited and voluntarily agreed to participate, and whose contact details (telephone numbers and email addresses) were provided by the Physiotherapy Council of Kenya.

Data were collected by the first author between August and December 2019, using a self-administered questionnaire. The attitude, perception and barrier sections of the EBP questionnaire by Jette et al. (2003), which had a Cronbach’s alpha score of 0.87, were used to collect the data.

Five Kenyan physiotherapists in academia, with a minimum qualification of a Master’s degree in physiotherapy, were requested to assess the content validity of the questionnaire. This was conducted using an iterative process until consensus was achieved.

This study questionnaire had a Cronbach’s alpha score of 0.751.

The first section of the tool dealt with demographic data that included survey items designed for physiotherapists involved in sports practice, while the second included a portion using a Likert scale format to capture attitudes and perceptions.

Nine survey items were provided to investigate attitudes to, and perceptions of, EBP. The four-point Likert scale, ranging from ‘strongly disagree’, ‘disagree’ and ‘agree’, to ‘strongly agree’, was later transformed into a simple scale of ‘agree’ and ‘disagree’.

The barriers to EBP were assessed in the third section and were rated from the ‘least important’ barrier to the ‘most important’ barrier.

The collected data were analysed using the IBM Statistics Version 25. Results were summarised into descriptive statistics and were displayed in tables and figures. During the analysis of attitudes, the scoring of negative statements was reversed to represent positive attitudes for those who disagreed. Associations between selected socio-demographic characteristics (gender, age, training, experience, specialisation) and attitudes, perceptions and barriers were determined using the Chi-square test. The level of significance was set at *p* ≤ 0.05.

### Ethical considerations

This study was approved by the Jomo Kenyatta University of Agriculture and Technology (JKUAT), reference number: JKU/2/4/896B. The participants gave informed consent for their participation, which was voluntary. They could also withdraw from the study at any time without suffering any repercussions.

## Results

### Socio-demographic information

**FIGURE 1:**
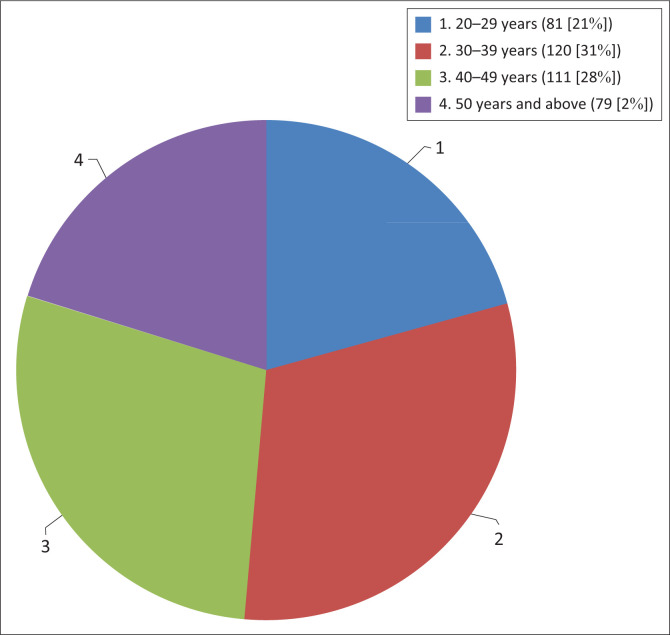
Age profile of the sample (*n* = 391).

A total of 391 (55.9%) of the 700 physiotherapists responded to the questionnaire. The majority of the participants were males (64.5%; *n* = 252) (Table 1). Most of the respondents were aged between 30 and 39 years (30.7%; *n* = 120) (Figure 1).

**TABLE 1:** Gender, experience, level of training and area of specialisation (*n* = 391).

Variable	Value	
	*n*	%
Gender		
Males	252	64.5
Females	139	35.5
Experience as a physiotherapist		
< 5 years	48	12.3
≥ 5–10 years	83	21.2
≥ 10–15 years	75	19.2
≥ 15–20 years	72	18.4
≥ 20 years	113	28.9
Level of physiotherapy training		
Diploma	238	60.9
B.Sc. students	22	5.6
B.Sc. graduates	84	21.5
M.Sc. students	27	6.9
M.Sc. graduates	6	1.5
Others: HND	11	2.8
PhD students	3	0.8
Area of specialisation		
Orthopaedics	13	16.6
Neuro	8	10.2
OMT	33	42.2
Musculoskeletal	5	6.3
Sports physiotherapy	7	8.8
Pain	1	1.2
Traumatology	1	1.2
Pulmonary therapy	1	1.2
Gynaecology	1	1.2
Cardiac rehabilitation	1	1.2
Non-core physiotherapy	8	10.2

HND, higher national diploma; OMT, orthopaedic manual therapy.

**FIGURE 2:**
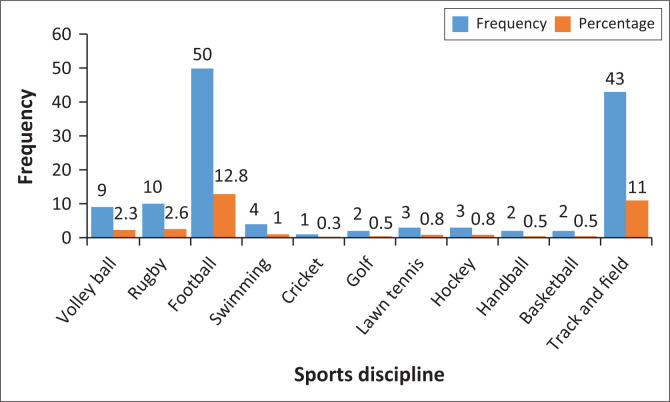
Sports discipline: Specific practices.

As seen in Table 1, those with > 20 years of work experience since graduating were in the majority (28.9%; *n* = 113). The majority of the respondents had received training at the diploma level (60.9%; *n* = 238), while the orthopaedic manual therapy (OMT) area of specialisation was the most common (42.2%; *n* = 33). The most frequently represented sports discipline was football (12.8%; *n* = 50), while the least represented discipline was cricket (0.3%; *n* = 1) (Figure 2).

### Attitudes and perceptions regarding evidence-based practice

As indicated in Tables 2 and Table 3, the respondents generally expressed positive attitudes to EBP. These were most evident in the agreement that ‘EBP is important so that the patients can receive the best possible treatment’ (95.9%; *n* = 375), and also in the agreement that it is important that ‘evidence-based guidelines relating to the work exist’ (84.6%; *n* = 331). Furthermore, as indicated in Table 2, there was strong disagreement with the statement that ‘there is not much point in conducting an EBP’ because there is ‘a lack of strong evidence supporting most of the work done’ (74.9%; *n* = 293) and with the statement that ‘the adoption of evidence-based practice places an unreasonable demand on physiotherapists’ (71.4%; *n* = 279).

**TABLE 2:** Attitudes and perceptions regarding evidence-based practice (EBP) (*n* = 391).

Attitudes and perceptions regarding EBP	Disagree		Agree	
	*n*	%	*n*	%
I consider it important that easily available evidence-based guidelines related to my work exist.	63	16.1	331	83.9
EBP is important so that the patients receive the best possible treatment.	16	4.1	375	95.9
The adoption of EBP places an unreasonable demand on physiotherapists.	279	71.4	112	28.6
EBP does not take into account the limitations of my day-to-day work.	221	56.5	170	43.5
EBP does not take into account my patients’ preferences.	241	61.6	150	38.4
There is not much point in doing EBP because there is a lack of strong evidence to support most of the work I do.	293	74.9	98	25.1
In making clinical decisions about my professional work, I value clinical field experiences more than literature from scientific studies.	211	54	180	46
Work-place experience is the most reliable way to know what really works.	194	49.6	187	50.4
Seeking relevant evidence from scientific studies is not very practical in the real world.	255	65.2	136	34.8

**TABLE 3:** Summary of attitudes and perceptions regarding evidence-based practice (EBP) (*n* = 391).

Attitudes towards EBP	*n*	*%*
Positive attitudes	370	94.6
Negative attitudes	21	5.4

Table 3 presents a summary of the perceived attitudes and perceptions regarding EBP. Those who agreed with more than half of the statements were deemed to hold positive attitudes towards EBP (94.6%; *n* = 370), while those who agreed with less than half of the statements were deemed to hold negative attitudes towards EBP (5.4%; *n* = 21).

### Association between demographic characteristics, other information, and attitudes and perceptions regarding evidence-based practice

As indicated in Table 4, there were no significant associations between the demographic characteristics (gender *p* = 0.104; age *p* = 0.495; training *p* = 0.0.590; work experience *p* = 0.980; and specialisation *p* = 0.649) and attitudes to, and perceptions of, EBP. The strength of association between the independent and outcome variables ranged from 0.010 to 0.109; that is small effect to large effect (see Table 4).

**TABLE 4:** Association between demographic characteristics and attitudes and perceptions regarding evidence-based practice (EBP) (*n* = 391).

Variable	Attitudes and perceptions towards EBP						Asymptotic significance (two-sided)		
	Negative attitude			Positive attitude			*χ* ^2^	*P*	*df*
	*n*	*%*	Eta	*n*	*%*	Eta			
**Gender**	-	-	0.082	-	-	0.082	0.104	2.638	1
Male (*n* = 252)	17	6.70	-	235	93.20	-	-	-	-
Female (*n* = 139)	4	2.80	-	135	97.10	-	-	-	-
**Age**	-	-	0.010	-	-	0.078	0.495	2.393	3
20–29 years (*n* = 81)	4	4.90	-	77	95.10	-	-	-	-
30–39 years (*n* = 120)	5	4.10	-	115	95.80	-	-	-	-
40–49 years (*n* = 111)	9	8.10	-	102	91.80	-	-	-	-
50 years and older (*n* = 79)	3	3.80	-	76	96.20	-	-	-	-
**Physiotherapy training (*n %*)**	-	-	0.012	-	-	0.109	0.590	4.644	6
Diploma (*n* = 238)	10	4.20	-	228	95.80	-	-	-	-
B.Sc. student (*n* = 22)	2	9.09	-	20	90.90	-	-	-	-
B.Sc. (*n* = 84)	6	7.14	-	78	92.80	-	-	-	-
M.Sc. student (*n* = 27)	3	11.10	-	24	88.88	-	-	-	-
M.Sc. (*n* = 6)	0	0.00	-	6	100.00	-	-	-	-
Others (Higher National Diploma) (*n* = 11)	0	0.00	-	11	100.00	-	-	-	-
Ph.D. student (*n* = 3)	0	0.00	-	3	100.00	-	-	-	-
**Work experience (*n %*)**	-	-	0.011	-	-	0.033	0.980	0.426	4
< 5 years (*n* = 48)	3	6.30	-	45	93.7	-	-	-	-
> 5 – < 10 years (*n* = 83)	5	6.10	-	78	93.9	-	-	-	-
> 10 – < 15 years (*n* = 75)	3	4.00	-	72	96	-	-	-	-
> 15 – < 20 years (*n* = 72)	4	5.50	-	68	94.4	-	-	-	-
> 20 years (*n* = 113)	6	5.30	-	107	94.6	-	-	-	-
**Specialisation in core physiotherapy areas *n* (%)**	-	-	0.023	-	-	0.023	0.649	0.207	1
Yes (*n* = 78)	5	6.40	-	73	93.6	-	-	-	-
No (*n* = 313)	16	5.10	-	297	94.8	-	-	-	-

*n*, number; Eta: *χ*^2^, chi-square; *P, p*-value; *df*, degrees of freedom.

Note: On account of rounding, none of the percentages adds up to 100%.

### Barriers to evidence-based practice

The barrier rated as the ‘most important barrier’ by majority of the participants (57.8%; *n* = 226) was ‘insufficient time’. Other barriers also ranked by many as ‘most important’ were ‘lack of generalisability of the literature findings to the sport patient population’ (56.3%; *n* = 220); ‘inability to apply the research findings to individual patients with unique characteristics’ (50.1%; *n* = 196); ‘limited ability to critically appraise the literature’ (47.6%; *n* = 186); and ‘lack of understanding of statistical analysis’ (45.8%; *n* = 179). As indicated in Table 5, ‘lack of interest’ was rated by most participants as the ‘least important’ barrier.

**TABLE 5:** Barriers to evidence-based practice (EBP) (*n* = 391).

Barriers to EBP	Least important		Less important		Slightly important		Important		Fairly important		Most important		Mean	Standard deviation
	*n*	%	*n*	%	*n*	%	*n*	%	*n*	%	*n*	%		
Insufficient time	38	10.0	23	5.9	45	11.5	24	6.1	34	8.7	226	57.8	4.71	1.775
Limited access to search engines and for interpreting evidence	41	10.5	43	11.0	38	9.7	29	7.4	68	17.4	172	44.0	4.42	1.801
Lack of research skills	113	28.9	36	9.2	27	6.9	38	9.7	26	6.6	151	38.6	3.72	2.141
Limited ability to critically appraise the literature	64	16.4	46	11.8	34	8.7	32	8.2	29	7.4	186	47.6	4.21	1.993
Lack of generalisability of the literature findings to my sporting patient population	42	10.7	33	8.4	31	7.9	32	8.2	33	8.4	220	56.3	4.64	1.822
Inability to apply research findings to individual patients with unique characteristics	48	12.3	40	10.2	41	10.5	31	7.9	35	9.0	196	50.1	4.41	1.884
Lack of understanding of statistical analysis	62	15.9	38	9.7	33	8.4	53	13.6	26	6.6	179	45.8	4.23	1.938
Lack of collective support among colleagues in my facility	86	22.0	48	12.3	30	7.7	29	7.4	36	9.2	162	41.4	3.94	2.075
Lack of interest	177	45.3	49	12.5	20	5.1	27	6.9	16	4.1	102	26.1	2.90	2.126
Lack of information resources	82	21.0	46	11.8	29	7.4	23	5.9	36	9.2	175	44.8	4.05	2.079

Note: Mean and standard deviation were calculated on the Likert scale of 1–6, where 1 = ‘least important’ and 6 = ‘most important’.

## Discussion

This study is the first to determine the attitudes, perceptions and barriers regarding EBP in sports physiotherapy in Kenya.

There were more male physiotherapists participants (64.5%) than females (35.5%) in this study. This largely mirrors the national gender distribution of physiotherapists in Kenya, with 56 % males and 44 % females (Physiotherapy council of Kenya, June, 2020). In Nigeria, Akinbo et al. (2008) recorded a gender distribution of 63% males and 37% females. However, Jette et al. (2003) in the USA; Salbach et al. (2009) in Canada; Heiwe et al. (2011) in Sweden and Ramírez-Vélez et al. (2015a) in Colombia, who conducted studies on EBP among physiotherapists, all found that female physiotherapists were in the majority, with more than 60% in each case.

Most of the respondents were aged between 30 and 39 years (30.7%), while the over-50 age group was the smallest. Owing to their training at college, the younger respondents would be expected to have positive attitudes and perceptions regarding EBP. This was a common finding in several studies (Jette et al. [2003] at 32.5%; Salbach et al. [2009] at 34.7%; and Heiwe et al. [2011] at 36.4%).

Most physiotherapists in Kenya trained at the diploma level (60.9%; *n* = 238), with minimal training in other areas at the postgraduate level. The differences in the levels of training did not significantly influence their attitudes to, and perceptions of, EBP. Regardless of their level of education and area of specialisation, the physiotherapists perceived EBP positively.

Most of the respondents (28.9%; *n* = 113) had over 20 years of work experience. Evidence-based practice is a relatively new concept in Kenya and therefore those with over 20 years of work experience might not have adequate information regarding EBP and might in fact require continuing professional training to enable them to implement the practice.

Among the physiotherapists, OMT was the most common specialisation, accounting for 42.2% of the specialities. Only one study (Jette et al., 2003) in the US, was found to have reported on the areas of physiotherapy specialisation. This study’s findings agreed with those of the current study, and an orthopaedics specialisation was significantly represented.

Our study established that 94.6% of the physiotherapists generally have positive attitudes towards EBP. However, no associations were found between the demographic characteristics of the respondents and their attitudes and perceptions regarding EBP. At this point, it should be noted that the number of participants with negative attitudes (*n* = 21) was found to be small compared to those with positive attitudes (*n* = 370) and the findings should be interpreted with caution.

Positive attitudes were also identified by Scurlock-Evans et al. (2014) in a systematic review, as well as in other studies which found physiotherapists with positive attitudes and perceptions regarding EBP (Frantz & Diener 2009; Hannes et al. 2009; Heiwe et al. 2011; Iles & Davidson 2006; Jette et al. 2003; Ramírez-Vélez et al. 2015b; Schreiber et al. 2009; Silva et al. 2015; Tadyanemhandu et al. 2016; Yahui & Swaminathan 2017). Specifically, the respondents were found to have a positive attitude to the fact that ‘evidence-based practice is important for offering patients the best possible treatment’; and that ‘it is important that evidence-based guidelines related to their work exist’. However, they disagreed with the statements that ‘evidence-based practice places an unreasonable demand on physiotherapists’ and that ‘evidence-based practice does not take into account patients’ preferences’. The authors considered the positive attitudes to the ideas with which they were presented as a positive foundation for building up an evidence-based, practise-conscious and friendly population of physiotherapists.

The barriers to evidence-based sports physiotherapy in Kenya are numerous and varied.

In this study, insufficient time was highlighted by 57.8% of the respondents as the ‘most important’ barrier. This echoes the perception that the ‘adoption of evidence-based practice places an unreasonable demand on physiotherapists’. This implies that, with the removal of the barriers or with some type of facilitation process, they would be willing to employ EBP at a higher level. This finding is also supported by systematic reviews which found insufficient time to be the most important barrier (Scurlock-Evans et al. 2014; Tadyanemhandu et al. 2016). Other studies also established that the respondents believed that insufficient time was the most important barrier: Salbach et al. (2007) at 52%; Akinbo et al. (2008) at 64%; Nilsagård and Lohse (2010) at 86%; Heiwe et al. (2011) at 43.5% and Ramírez-Vélez et al. (2015a) at 84%.

In the context of time being a barrier, the authors consider it important for healthcare institutions to create EBP-friendly incentives, such as the provision of internet-connected computers with access to the necessary search engines and databases. This can be justified by the perception that: ‘I consider it important that easily-available evidence-based guidelines related to my work exist’. This would probably encourage the physiotherapists to allocate time for searching for evidence. Thereafter, the improved quality of care as a result of EBP could possibly reduce patient volumes in the clinics. Institutions may also find it valuable to borrow from Iles and Davidson’s (2006) findings, which note that the usage of high-quality and pre-appraised evidence from the PEDro and Cochrane databases reduced the time necessary to apply EBP processes, even if the clinicians were very busy.

This study found that 56.3% of the respondents reported a lack of generalisability of the literature findings to the sporting patient population (patients with specific conditions) as a ‘most important’ barrier. Owing to insufficient empirical studies in the Kenyan context, the authors found this concern to be of importance, warranting more research pertaining to locally identified problems in Kenya.

The current study found that 50.1% of the respondents rated the inability to apply research findings to individual patients with unique characteristics as a ‘most important’ barrier. To a lesser degree, other researchers also found this inability to apply research findings to individual patients with unique characteristics as a barrier. For instance, in Nigeria, Akinbo et al. (2008) reported a 37% barrier rating; while in Colombia, Ramírez-Vélez et al. (2015b) reported 41%. The authors recognise that failure to apply findings may be multifaceted. Therefore, to establish whether these are knowledge-based or resource-based limitations, or perhaps go beyond this, would call for further investigation.

In this study, 47.6% of the respondents rated the inability to critically appraise the literature or the evidence as a ‘most important’ barrier. This was rated lower in other countries and was considered less of a barrier in the US (Jette et al. 2003) at 20%; in Sweden (Heiwe et al. 2011) at 32%; and in Colombia (Ramírez-Vélez et al. 2015b) at 10.7%. It is therefore evident that physiotherapists in Kenya experience difficulties in critically appraising the literature. The authors found this to be related to their knowledge. It could be overcome through the implementation of continuing professional development initiatives.

A lack of understanding of statistical analysis and of information resources was highlighted by 45.8% and 44.8% of the respondents as a ‘most important’ barrier, respectively. A study in Colombia (Ramírez-Vélez et al. 2015a) established that 53% of the respondents considered lack of understanding of statistical analysis as a barrier. Furthermore, a study in Sweden (Heiwe et al. 2011) found that 33%, and one in Canada (Salbach et al. 2009) found that 36.4%, of their respondents had identified an inability to understand statistical data or analysis as a barrier. A lack of information resources (41%) was identified in a Nigerian study as an important barrier (Akinbo et al. 2008). Along with the exposure necessary to other areas of EBP, Kenya physiotherapists would also require analytical skills to consider all forms of data. This would position them better to interpret the findings they, or other secondary sources generate. To provide this support, institutions would need to procure a wide range of databases, capable of providing recent peer-reviewed and relevant material as the need arises.

Limited access to search engines and the interpretation of the evidence was identified by 44% as a ‘most important’ barrier. In Nigeria, Akinbo et al. (2008) reported that 51% of the respondents had access to online databases at the workplace, while 64% had access to paper journals. In the US, Jette et al. (2003) reported that 30% of the study respondents had access to online databases at the workplace, while less than 10% were able to access online databases away from the workplace. Since the physiotherapists studied in this research had positive attitudes towards EBP, it would be worthwhile to use this goodwill to encourage healthcare institutions to invest in the provision of databases, while rewarding the physiotherapists for using the same.

In this study, lack of research skills was identified as a ‘most important’ barrier by only 38.6% of the respondents. This barrier was more important in other studies: 56% in Ramírez-Vélez et al.’s (2015b) study in Colombia. Considering that physiotherapy training includes instruction in research methods at all levels, although at varying levels of complexity, the physiotherapists involved in this research were not expected to rate the lack of such skills very highly. However, it would be important to up-scale the research skills of all the physiotherapists over time, in order for them to remain competent during any efforts to execute EBP.

In the current study, ‘lack of interest’ was viewed by a large number of respondents (45.3%) as a ‘least important’ barrier. This was similarly reflected when the participants responded to the question of attitude, which indicated that ‘evidence-based practice is important so that the patients receive the best possible treatment’. In studies in Nigeria (Akinbo et al. 2008) and the USA (Jette et al. 2003) it was found that the study participants did not view lack of interest as a barrier. This was interpreted by the authors as an expression of interest, particularly in the Kenyan context. The authors, however, have noted which barriers were identified and recommend to stakeholders in sports physiotherapy training and practice that those barriers be addressed. The associated positive attitudes and perceptions regarding EBP will serve to facilitate the adoption of the type of programmes and training to be employed.

## Recommendations

The study recommends the following:

- Healthcare institutions that attend to clients with sports injuries should provide electronic access to databases, and the physical infrastructure to support EBP.
- Continuing professional development should enhance competencies in data analysis, particularly in skills such as meta-analysis.
- More studies should be implemented to explore in-depth realities related to the barriers highlighted.

## Conclusion

This study concludes that, generally, physiotherapists have a strongly positive attitude towards EBP and the authors are of the opinion that EBP is important in that it allows patients to receive the best possible treatment. The study further endorses the fact that easily available evidence-based guidelines related to work do, in fact, exist. The fact that level of education or specialisation were not found to influence physiotherapists’ attitudes to EBP, negatively points to a population that would use knowledge empowerment positively to transform the quality of sports injury care in Kenya. The main barrier identified in this study was insufficient time. However, other barriers critical to the development of EBP standards include a lack of generalisability of the literature findings to the sporting patient population. Lack of interest in EBP was considered least important by the majority of the respondents.
